# The prevalence of Chlamydia trachomatis in patients who remained symptomatic after completion of sexually transmitted infection treatment

**Published:** 2013-04

**Authors:** Maryam Afrakhteh, Atossa Mahdavi, Hadi Beyhaghi, Afshin Moradi, Sima Gity, Shirin Zafargandi, Zahra Zonoubi

**Affiliations:** 1 *Department of Obstetrics and Gynecology, Shohadaye Tajrish Hospital, Shahid Beheshti University of Medical Sciences, Tehran, Iran.*; 2 *Department of Obstetrics and Gynecology, Shariati Hospital, Tehran University of Medical Sciences, Tehran, Iran. *; 3 *Center for Clinical Excellence, Taleghani Hopital, Shahid Beheshti University of Medical Sciences, Tehran, Iran.*; 4 *Cancer Research Center, Shahid Beheshti University of Medical Sciences, Tehran, Iran.*; 5 *Department of Obstetrics and Gynecology, Mahdieh Hospital, Shahid Beheshti University of Medical Sciences, Tehran, Iran.*

**Keywords:** *Chlamydia trachomatis*, *Differential diagnosis*, *Sexually transmitted infection*

## Abstract

**Background:** Sexually transmitted infections (STIs) are among the most common causes of illness in the world and have far-reaching health, economic and social consequences for many countries. Failure to diagnose and treat STIs at an early stage may result in serious complications and sequels.

**Objective:** This study aimed to determine the prevalence of Chlamydia trachomatis infection in patients who remain symptomatic after completion of their first episode of treatment for STI.

**Materials and Methods:** We conducted a cross-sectional study on 49 patients suffering from symptoms or signs of sexually transmitted infections despite their first complete anti STI treatment. Conducting physical exam and smear preparation from vaginal discharge, diagnosis was confirmed by Polymerase chain reaction (PCR) method on every patient’s first-voided urine sample.

**Results:** Among the etiologic factors investigated in this study, Chlamydia was reported in 17 patients. Trichomoniasis, Candidiasis, Gonorrhea and nonspecific germs were next organisms with 11, 9, 6 and 6 patients, respectively. Sixteen specimens were PCR positive (32.65%), while 33 patients had negative PCR results (67.34%) for Chlamydia trachomatis.

**Conclusion:** Gonorrheal infection was the most prevalent infection in patients with completed treatment (6/10), which must be remembered in patients follow ups, because this prevalence warrants empirical therapy for Gonorrheain similar clinical conditions. Chlamydia trachomatis was the responsible organism in approximately a quarter of patients (17/75) who despite their full compliance on anti-Chlamydial treatment still suffered from signs and symptoms of STI. This rate also recommends empirical therapy for Chlamydia trachomatis in the similar clinical signs and symptoms.

## Introduction

Sexually transmitted infections (STIs) are among the most common causes of illness in the world and have far-reaching health, economic and social consequences for many countries (1). 

Failure to diagnose and treat STIs at an early stage may result in serious complications and sequels. Sexual and reproductive tract infections other than human immunodeficiency viruses (HIV) are important global health priorities, particularly *Chlamydia* and *gonorrhea* with disease burden like 7 million Disability adjusted life years (DALYs). *Chlamydia trachomatis* is the most common bacterial sexually transmitted disease (STD) in the United States (2-4). Chlamydial infection causes significant sequels in women which are responsible for most costs attributable to the infection. Pelvic inflammatory disease is the common complication of Chlamydial infection and accounts for a sizable proportion of the health care costs. 

In addition, scarring caused by the inflammation of Chlamydial infection leads to high rates of long term complications, including infertility, ectopic pregnancy, and chronic pelvic pain. Chlamydial infection has also been associated with an increased risk for transmission of *HIV* (5). In order to prevent the sequels of Chlamydial infection, initial transmission must be prevented or infected people must be identified and treated before the occurrence of complications (6). 

Early identification of infected cases is difficult mostly because symptoms associated with Chlamydial infection are nonspecific (7). Furthermore, infections may persist for several months to years in untreated women (8). Etiological diagnosis of STIs including *Chlamydia trachomatis* is difficult for health care providers in many settings. In addition, the sensitivity and specificity of commercially available tests can vary significantly, adversely affecting the reliability of laboratory testing for STI diagnosis (9). 

Health care providers often face patients who have been treated for STI in different health care settings and still suffer from previous signs and symptoms in spite of the treatment. This might be due to reasons such as poor compliance, inaccurate diagnosis, improper treatment, and recurrence or re-infection due for example to untreated partner. Making an accurate diagnosis is therefore, the cornerstone of attaining desirable clinical outcomes. 

In the context of evidence based medicine, determining the pretest probabilities of different etiological causes serves a crucial role in final diagnosis. Pretest probability that exceeds treatment threshold (the probability of infection above which empirical therapy is indicated) can even make further diagnostic measures unnecessary (10). 

In continues with our previous research about STIs, this study aimed to determine the prevalence of *Chlamydia trachomatis* infection as a differential diagnosis of STI in patients who remain symptomatic after completion of their prior treatment for STI (11).

## Materials and methods

**Case ascertainment**

In this cross-sectional study, using randomized cluster sampling five centers were selected from all health centers with laboratory facilities affiliated to Shahid Beheshti University of Medical Sciences. These centers provide nearly free of charge primary health services actively throughout their catchment area. The research team provided standard training to health care providers concerning the diagnostic and therapeutic protocols for STIs based on WHO STI guidelines (6, 12). A total of 301 consecutive female patients entered the study between May 2005 till May 2008. 

Patients with complaints including discharge, pain, pruritus or redness of the external genitalia or pain and irritation in the urinary tract, who had received no prior antimicrobial treatment in the last 4 weeks, were considered. After history taking and physical examination, smear preparations were made from cervical secretions. Based on the predefined diagnostic criteria (Table I) patients were categorized in 5 groups. In doing so, the etiologic factors were determined and treatments both for patients and their spouses were commenced accordingly. Mixed infections were excluded.

**Figure 1:**
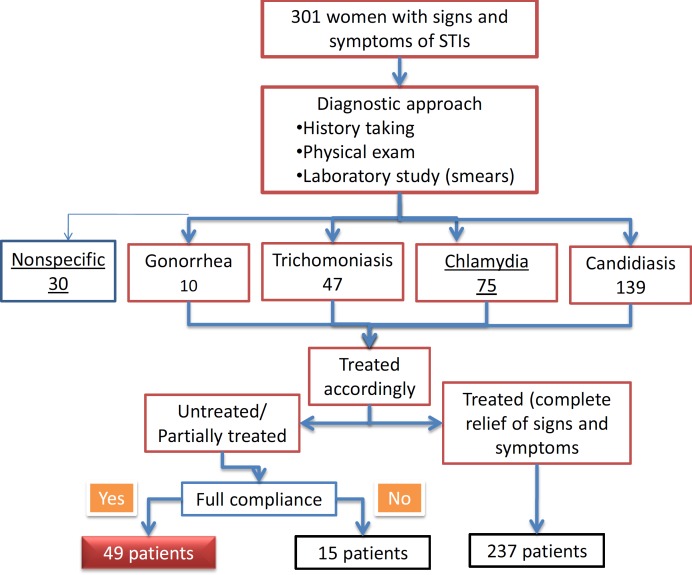
Case ascertainment

During their follow up visit, three weeks after the completion of treatment, among 301 patients, 237 women were fully treated in terms of the relief of signs and symptoms. Those who still suffered from STI signs and symptoms were interviewed to determine their compliance. Alongside checking their drug blister packs, patients and their spouses were asked about the completion of medication; also, concurrent use of other drugs was determined to rule out drug interaction. Among 64 patients who remained symptomatic despite receiving treatment, 49 patients who had fully complied with the treatment regimen were enrolled in our study (Figure 1).

**Study protocol**

The enrolled patients were referred to Shohadaye Tajrish Hospital, a university medical center, for further evaluation and treatment, where patients once again underwent the process of history taking, physical examination and smear preparation by senior residents of obstetrics and gynecology department. Three smears including one for gram staining (to detect *gonococcus* and *candida*), one for Giemsa staining (to detect *Chlamydia*), and a Wet Mount smear (to detect *Trichomonas*) were taken from each patient and were sent to pathobiology laboratory of the hospital. The pathologic diagnosis was made only by the hospital pathologist. In addition, all patients’ first-voided urine specimens were sent to university Polymerase Chain Reaction (PCR) Center in Shahid Beheshti School of Medicine to detect the presence of *Chlamydia trachomatis*. PCR method is as the following: Ten milliliter of urine for each patient was pooled and centrifuged at 1200 rpm for 10 min. Lysis buffer (SDS 10%, Tris-EDTA 50mM, PH=8) containing proteinase K (0.1mg/ml) was added to the sediment and was incubated in 60^o^C. 

Equivalent to the volume of the sediment; firstly phenol then chloroform were added. Isoproponalol was added to the supernatant and was stored at -20^o^C for a night. Then, after washing with alcohol 70%, DNA sediment was dispersed in distilled water. After DNA extraction, its concentration and quality was measured by Ultrspect 3000 and DNA (5µL) was used as template.

The PCR mixture contained 10 pmol of each primer, 1.5 units of Taq DNA polymerase (QIAGN) 200m M dNTPs, 2mM Mgel2 in a final volume of 25 µL. The PCR conditions consisted of one step 95^o^C for 5 minutes, 35 cycles of 94^o^C for 45 seconds, 59^o^C for 45 seconds, 72^o^C for 45 seconds and final extension at 72^o^C for 10 minutes. The PCR product were analyzed on a 2.5% agarose gel. The thermal cycler was ASTEC PC320. Primers were: Pf: CCT/GTG/GGG/AAT/ CCT/GCT/ GCT/GAA and r: GTC/GAA/AAC/ AAA/GTCATCCAGTA/GTA. 

Informed written consent was taken from all the participants before initiating the interview. The study protocol was approved by the ethical committee of the Urology Research Center-affiliated to Shahid Beheshti University of Medical Sciences. 

**Statistical analysis**

Data was collected via questionnaires and forms. It was analyzed by Epi-Info software version 3.4.1. Prevalence of every infection was calculated. 

## Results

Among 49 female patients enrolled in this study 42 were married (85.71%). Participants aged 15-45 years with the mean±SD of 28.83±7.76 years. Oral Contraceptive Pills (OCP) was the most common method of contraception (42.73%), and withdrawal method (31.57%) comes next following by barrier methods (23.64%). Vasectomy and tubal ligation (TL) constituted less than 2.04% of contraceptive methods. Ninety five percent of patients were living in urban areas and the majority of them (66.73%) were categorized as middle socio-economic class.

**Figure2:**
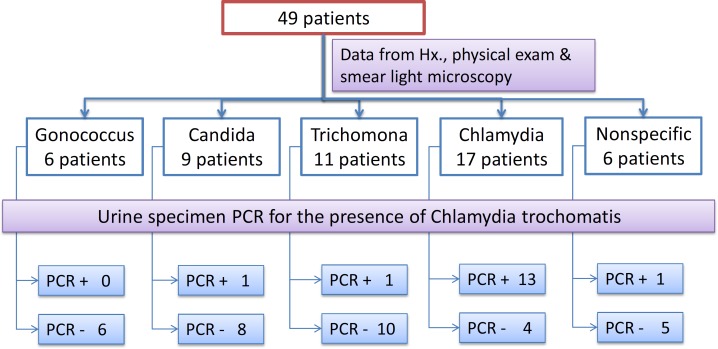
STI differential diagnoses of enrolled patients

Based on the history, physical exam and smear findings, participants primarily fell in 5 diagnostic groups according to predefined diagnostic criteria (Table I). Among the etiologic factors investigated in this study, *Chlamydia* was reported in 17 (17 out of 75 or 23%) patients. Trichomoniasis, Candidiasis, Gonorrhea and nonspecific germs were the next organisms with 11 (11 out of 47 or 23%), 9 (9 out of 139 or 6%), 6 (6 out of 10 or 60%) and 6 (6 out of 30 or 20%) patients, respectively. It means that Gonorrhea was the most prevalent organism after anti gonorrheal treatment. *Chlamydia and *Trichomona infections were the next prevalent persistent infections. Conducting PCR on first-voided urine samples of all 49 patients as a confirmatory test for *Chlamydia trachomatis* revealed that 16 specimens were PCR positive (32.65%), while 33 patients had negative PCR results (67.34%) (Figure 2).

**Table I:** Diagnostic criteria for identifying STI causative pathogens (1), (9)

**Infection**	**Diagnostic criteria**
**Neisseria gonorrhoeae**	Cervical purulent discharge, irregular vaginal bleeding, pelvic pain, morning drop for men, finding gram negative *diplococcus *in the smear
**Trichomona**	Foamy discharge, red and inflamed vaginal mucosa, pain, pruritus, and motile *T. vaginalis *which are usually identified easily in the saline specimen (wet mount)
**Candida**	Pruritus, vaginal soreness, dyspareunia, external dysuria, and abnormal white vaginal discharge. The yeast or *pseudohyphae *are identified in the KOH specimen or visualized as long, round and oval shaped gram positive organisms on gram staining.
**Chlamydia**	Cervical erosions with copious mucopurulent discharge, and testing urine or swab specimens collected from the endocervix or vagina in women and testing urethral swab specimen in men.
**Non specific **	Malodorous discharge, no dyspareunia, adherent discharge, saline microscopy PMN: EC <1; loss of rods; increased coccobacilli; clue cells (>90%).

**Figure 3:**
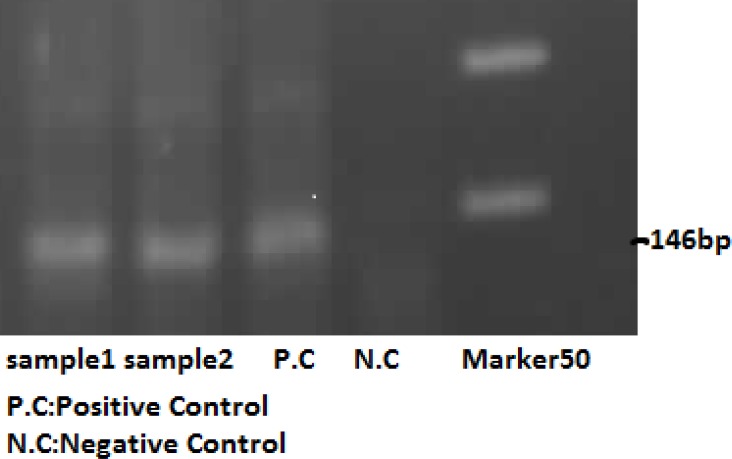
PCR method

## Discussion

The findings of this study show that among 49 enrolled patients in this study, 16 subjects had been diagnosed with *Chlamydia trachomatis* infection according to urine sample PCR as a diagnostic gold standard in this study. Among them, 13 cases were previously diagnosed with chlamydial infection based on composite criteria of history, physical exam and smear light microscopy. 

The diagnostic accuracy of these composite criteria is rather low compare to first-voided urine sample PCR with sensitivity and specificity of 92.3% and 98.6% respectively (13-15). Thus, among 17 patients who have previously diagnosed with *Chlamydia trachomatis, *urine sample PCR confirmed the diagnosis of 13 cases which is roughly in agreement with the previously reported specificity of 80% for the diagnostic criteria (16).

*Chlamydia trachomatis *is one of the most common etiologic factors for sexually transmitted infections (17). Various studies have been carried out to determine the prevalence of Chlamydial infection in different countries. The reported prevalence of Chlamydia trachomatis infection in women of reproductive age lies between 3-13% in different studies (5, 17-23). In Iran, however, the prevalence of *C. trachomatis* infection appears to be higher in various age groups, from 12-28% in different studies (24-29). Chamani *et al* investigated the prevalence of *Chlamydia* infection in 1502 women aged 15-42 who attended Obstetrics and Gynecology clinics in Tehran by conducting PCR on DNA extracted from first-voided urine specimen. In their study 133 patients (12.6%) were PCR positive for Chlamydia trachomatis (30). 

In the similar study, Bakhtiari *et al* studied the prevalence of Chlamydia trachomatis infection among 550 sexually active women <45 years, attending gynecology clinics in Babol, Iran. The prevalence of C. trachomatis was 11.6% and it was more common among women under 25 years (31). A recent study by Jenab *et al* suggests that among 80 women aged between 20 and 60 years, who visited in an urban outpatient gynecology clinic in Isfahan, Iran, 17 patients (21.15%) were infected with Chlamydia. 

This study confirmed the presence of Chlamydia trachomatis with conducting PCR on cervical swab specimens (32). Nevertheless, current study, for the first time, measured the prevalence of *Chlamydia trachomatis* infection in patients suffering from signs and symptoms of sexually transmitted infections despite receiving full course of treatment. Higher rate of *Chlamydia trachomatis* infection in this study can be primarily attributable to sampling frame and characteristics of enrolled subjects. Public health models show that when disease prevalence is high in a population, the diagnostic and treatment threshold should be low (10). 

It is prudent in circumstances of high disease prevalence to over treat the population at risk, so as not to miss subclinical cases, because once the patient leaves the clinic without proper therapy, it can be difficult to locate her for timely referral. Without proper treatment, the patient endangers not only herself, but her sexual partners as well (33). It seems that high pretest probability of *C. trachomatis* infection in patients who suffer from STI signs and symptoms despite fully complied prior treatment make any further diagnostic measure for detection of *Chlamydia trachomatis* unnecessary. 

There are certain limitations in this study; including the accuracy of diagnostic facilities particularly in the case ascertainment phase, which was tried to be overcome by utilizing a combination of history, physical exam and laboratory findings. Also for Gonorrheal infections accuracy of culture methods was more desirable. Since the study sample was chiefly consisted of urban population of selected areas in Tehran, the sampling frame is another challenge in the present study and might cause concern regarding the generalization of the results. 

Besides, the results are limited since most people seek STD care from private practitioners, while this study was mainly centered on patients with sexually transmitted disease (STD) who attend public clinics (34). In addition, up to 80% of female patients with urogenital chlamydial infection are asymptomatic especially in, while in this study we chiefly focused on symptomatic cases (35). 

Finally, only 13 patients were PCR positive in chlamydial group, and 3 PCR positives were distributed equally in trichomoniasis, candidiasis and nonspecific germs; which could be attributed to lower accuracy of the combined diagnostic tests.

## Conclusion

Gonorrheal infection was the most prevalent infection in patients with completed treatment (6 out of 10), which must be remembered in patients follow ups. Because this prevalence warrants empirical therapy for Gonorrheain the similar clinical conditions. On the other hand, *Chlamydia trachomatis* was the responsible organism in approximately a quarter of patients (17 out of 75) who despite their full compliance on anti-Chlamydial treatment still suffered from signs and symptoms of STI. Considering the high rate of Chlamydia trachomatis infection in this patient group, empirical therapy of all similar patients for Chlamydial infection seems to be warranted in Iranian health care system.
